# Temporal Stability of Bacterial Communities in Antarctic Sponges

**DOI:** 10.3389/fmicb.2019.02699

**Published:** 2019-11-22

**Authors:** César A. Cárdenas, Alejandro Font, Georg Steinert, Rodolfo Rondon, Marcelo González-Aravena

**Affiliations:** ^1^Departamento Científico, Instituto Antártico Chileno, Punta Arenas, Chile; ^2^Institute for Chemistry and Biology of the Marine Environment, Carl von Ossietzky University of Oldenburg, Oldenburg, Germany

**Keywords:** Porifera, cold-water sponges, Antarctica, WAP, 16S rRNA, environmental variability

## Abstract

Marine sponges host dense, diverse, and species-specific microbial communities around the globe; however, most of the current knowledge is restricted to species from tropical and temperate waters. Only recently, some studies have assessed the microbiome of a few Antarctic sponges; however, contrary to low mid-latitude sponges, the knowledge about temporal (stability) patterns in the bacterial communities of Antarctic sponges is absent. Here, we studied the temporal patterns of bacterial communities in the Antarctic sponges *Mycale* (*Oxymycale*) *acerata*, *Isodictya* sp., *Hymeniacidon torquata*, and *Tedania* (*Tedaniopsis*) *wellsae* that were tagged *in situ* and monitored during three austral summers over a 24-month period. By using amplicon sequencing of the bacterial 16S rRNA gene we found that the microbiome differed between species. In general, bacterial communities were dominated by gammaproteobacterial OTUs; however, *M. acerata* showed the most distinct pattern, being dominated by a single betaproteobacterial OTU. The analysis at OTU level (defined at 97% sequence similarity) showed a highly stable bacterial community through time, despite the abnormal seawater temperatures (reaching 3°C) and rates of temperature increase of 0.15°C day^–1^ recorded in austral summer 2017. Sponges were characterized by a small core bacterial community that accounted for a high percentage of the abundance. Overall, no consistent changes in core OTU abundance were recorded for all studied species, confirming a high temporal stability of the microbiome. In addition, predicted functional pathway profiles showed that the most abundant pathways among all sponges belonged mostly to metabolism pathway groups (e.g., amino acid, carbohydrate, energy, and nucleotide). The predicted functional pathway patterns differed among the four sponge species. However, no clear temporal differences were detected supporting what was found in terms of the relatively stable composition of the bacterial communities.

## Introduction

Marine sponges (Phylum Porifera) are considered reservoirs of exceptional microbial diversity, constituting a major contributor to the total prokaryotic diversity of the world’s oceans (Thomas et al., 2016). Diverse and abundant communities have been described from shallow- and deep-water sponges across the world (Taylor et al., 2007; Schöttner et al., 2013; Reveillaud et al., 2014; Rodríguez-Marconi et al., 2015; Thomas et al., 2016; Steinert et al., 2019). As sessile filter-feeders, sponges are constantly interacting with microbes in the surrounding seawater; however, they harbor distinct symbiotic microbial communities (Thomas et al., 2016; Pita et al., 2018). Microbial symbionts play a key role for the health and survival of sponges and are thought to play functional roles including nutrient cycling, vitamin production, photosynthesis, and production of secondary metabolites (Taylor et al., 2007; Fan et al., 2012; de Goeij et al., 2013; Webster and Thomas, 2016; Botté et al., 2019). Functional analyses of sponge-associated microbes proposed a broad range of unique and shared genomic features of sponge symbionts, including the metabolism of carbon, nitrogen, or sulfur, the biosynthesis of amino acids and cofactors, cellular stress responses, or motility and adhesion (Webster and Thomas, 2016; Fan et al., 2012).

According to the abundance of bacterial symbionts, sponges have been classified as high microbial abundance (HMA) and low microbial abundance (LMA) (Vacelet and Donadey, 1977; Hentschel et al., 2003; Gloeckner et al., 2014). This classification incorporates distinctions not only in terms of abundance, but also diversity, specificity, as well as host physiology and morphology (Hentschel et al., 2003; Weisz et al., 2008; Schläppy et al., 2010; Moitinho-Silva et al., 2017). With some exceptions (e.g., Cao et al., 2012; White et al., 2012), both HMA and LMA sponges tend to exhibit a high degree of host specificity and stability (Easson and Thacker, 2014; Erwin et al., 2015; De Mares et al., 2017). Several studies have assessed the stability in sponge microbiomes when exposed to changes in environmental factors such as temperature, nutrient availability, light, and sedimentation (Friedrich et al., 2001; Lemoine et al., 2007; Webster et al., 2008; Simister R. et al., 2012; Simister R. L. et al., 2012; Fan et al., 2013; Cárdenas et al., 2014; Pineda et al., 2016, 2017; Weigel and Erwin, 2017). While some sponge-associated microbiomes tend to be highly stable when exposed to stress (Simister R. et al., 2012; Luter et al., 2014; Pineda et al., 2016), other studies have reported shifts and even disruption of the microbiome (see Lemoine et al., 2007; Webster et al., 2008; Fan et al., 2013; Ramsby et al., 2018).

The temporal stability in the sponge microbiome has been previously assessed in sponges from different latitudes (e.g., Björk et al., 2013; Hardoim and Costa, 2014; Erwin et al., 2015). A few studies have monitored the same individuals over time (Anderson et al., 2010; Erwin et al., 2012), providing new insights on variation in the microbiome over time and individual variation among hosts. Repeated sampling of individuals can also provide important baseline information on natural levels of symbiont variability and responses to abnormal environmental variation (Erwin et al., 2012).

In contrast to the vast amount of knowledge on sponge-symbionts from tropical and temperate latitudes, data on microbial communities associated with Antarctic sponges, which are important benthic components (Dayton et al., 1974; Cárdenas et al., 2016), are still scarce. Available information is limited for a few species from McMurdo Sound, East Antarctica, and some others from King George Island, South Shetland Islands (Maritime Antarctica), and Doumer Island, Palmer Archipelago in the Western Antarctic Peninsula (WAP) (Webster et al., 2004; Rodríguez-Marconi et al., 2015; Cárdenas et al., 2018a; see Lo Giudice and Rizzo, 2018 for review; Steinert et al., 2019). Results from these studies have shown that Antarctic species host diverse bacterial communities that are mainly dominated by Proteobacteria and Bacteroidetes, suggesting they might correspond to LMA sponges (Rodríguez-Marconi et al., 2015; Cárdenas et al., 2018a; Steinert et al., 2019).

The WAP is one of the areas that has been undergoing profound changes in recent decades, due to rapid environmental change (Cook et al., 2016; Meredith et al., 2017), with warming of the upper layers of the shelf exceeding 1°C (reviewed by Moffat and Meredith, 2018). Winter shallow seawater temperatures around the WAP are relative similar regionally, being close to −1.8°C. In contrast, summer temperatures show some spatial variability, reaching peaks between 1.5 and 2°C in Marguerite Bay and Anvers Island (Barnes et al., 2006; Peck, 2018). Recent findings have reported significant short-term increases in seawater temperature in South Bay, Doumer Island, Palmer Archipelago (Cárdenas et al., 2018b), reaching 3°C with warming rates of 0.15°C per day.

Previous studies on other Antarctic invertebrates suggested that many species are not well adapted to warming, being highly sensitive to small variability in water temperature (reviewed by Peck, 2018). Considering that even small variations in environmental condition can have significant effects on the physiology of some organisms (Morley et al., 2010), the increase in environmental variability and warming seawater could affect the stability of the microbial communities associated with Antarctic sponges, hence potentially affecting the functional roles of the whole sponge holobiont in a cascading ecosystem framework (Pita et al., 2018). Research on tropical and temperate species have reported shifts in the microbial communities of some species (Lemoine et al., 2007; Webster et al., 2008; Simister R. L. et al., 2012) and in some cases warming affects microbial function (Lopez-Legentil et al., 2010; Fan et al., 2013). However, despite a few studies characterizing associated microbial communities on cold-water species (Webster et al., 2004; Schöttner et al., 2013; Rodríguez-Marconi et al., 2015; Cárdenas et al., 2018a), no studies on the effect of warming on the bacterial communities of cold-water sponge species are available to date.

Recent evidence contradicts the paradigm that Antarctic sponges live in the slow lane, as some Antarctic sponges can respond rapidly to environmental changes such as collapse of ice shelves (Fillinger et al., 2013) or shifts in oceanographic regimes (Dayton et al., 2016). In this regard, greater-than-expected phenotypic plasticity of metabolic physiology in some Antarctic demosponges suggest they are better able to respond to seasonal and extreme environmental change, which may be linked to their symbiotic communities (Morley et al., 2016, 2019). However, this assumption still needs to be confirmed. Our knowledge on the composition and roles of the microbiome of Antarctic sponges is still scarce. Here we monitored the bacterial communities associated with four Antarctic sponge species that were tagged *in situ* and sampled during three austral summers over a 24-month period from February 2016 to February 2018 in shallow-water rocky reefs at Doumer Island, Palmer Archipelago, WAP. We aimed to determine the temporal patterns in diversity and structure in the bacterial communities of four studies species over time, also assessing the effect of changes in water temperature and potential negative effects on the stability of their associated microbes. We also predicted functional pathway profiles from metagenomic 16S rRNA gene data to investigate potential temporal functional patterns between and within the four sponge species using the Tax4Fun functional gene profile prediction software with sponge bacterial community 16S rRNA gene data as input. In addition, we applied a Tax4Fun analysis to the core bacterial OTU and predicted functional pathway profiles from an OTU table grouped into following sponge-specific community types: (a) core (i.e., OTUs present in all host-specific samples), (b) shared (i.e., OTUs present in two different years within each sponge host), and (c) transient (i.e., OTUs only present in one single year). Nevertheless, the predicted functional profiles remain to be verified by metagenomic or whole genome sequencing in future sponge microbiome research. Despite this, predictive functional profiling can serve as a complementary and cost-effective metagenomic pre-study.

## Materials and Methods

### Sample Collection

**FIGURE 1:**
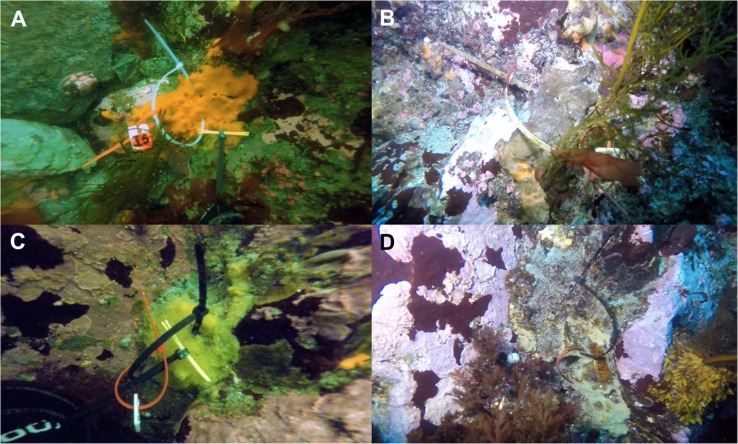
Examples of tagged specimens of Antarctic sponges monitored during three austral summers over a 24-month period (2016–2018) at Doumer Island, Palmer Archipelago, WAP. **(A,B)** *Isodictya* sp. (Iso.1) and **(C,D)** *Hymeniacidon torquata* (Hym.1) specimens sampled in 2016 and 2018, respectively.

A total of 60 samples of the sponges *Mycale (Oxymycale) acerata* (Order Poecilosclerida, *n* = 6), *Isodictya* sp. (Order Poecilosclerida, *n* = 26), *Hymeniacidon torquata* (Order Suberitida; *n* = 23), and *Tedania* (*Tedaniopsis*) *wellsae* (Order Poecilosclerida, *n* = 5) were tagged by SCUBA divers at 10 m depth in February (austral summer) 2016, at Cape Kemp in Doumer Island, Palmer Archipelago, WAP (64°51′58.6″S; 63°37′46.7″W). Sponge individuals were sampled and tagged *in situ* with plastic cable ties and numbered plastic tags during February 2016. Then the study site was monitored during February 2017 and January 2018, and individuals found were resampled to monitor their bacterial communities over time (Figure 1). Sampling was conducted only during summer months because of challenges associated with Antarctic research such as limited access due to difficult operating environment during most part of the year (Kennicutt et al., 2016). Tissue samples were processed at the laboratory of Yelcho Scientific Station (INACH) and were then preserved in plastic tubes with RNALater© and kept at −20°C until transported to Laboratorio de Biorrecursos INACH, Punta Arenas (Chile), for subsequent DNA extraction.

Seawater temperature was recorded with three HOBO pendant temperature data loggers (Part #UA-002-XX; Onset Computer Corp.). Measurements were recorded every 2 h [see Cárdenas et al. (2018b) for details]. Data loggers were deployed in January 2016 and recovered and redeployed in February 2017. In January 2018, only one data logger was recovered; however, it was damaged by icebergs, a new one was then deployed for the sampling period in summer 2018.

### DNA Extraction and Sequencing

Genomic DNA from sponge tissue (≈0.3 g) for 16S rRNA gene amplicon sequencing was extracted using the Precellys© Evolution homogenizer with a Cryolys cooling unit (Bertin Technologies, Montigny-Le-Bretonneux, France) and a DNeasy PowerSoil Kit (Qiagen, Hilden, Germany), following the manufacturer’s instructions. Extractions were performed using both internal and external sponge tissue in order to obtain the whole bacterial community. DNA concentrations were measured using a Nanoquant Spectrophotometer (Tecan, Switzerland). Approximate 500 ng of DNA sample were sent to the Dalhousie University CGEB-IMR^1^ for V4–V5 rRNA gene library preparation and sequencing. Primers used correspond to 517f GTGYCAGCMGCCGCGGTAA and 926r CCGYCAATTYMTTTRAGTTT (Walters et al., 2015). Samples were multiplexed using a dual indexing approach and sequenced using an Illumina Miseq with paired-end 300 + 300 bp reads. All PCR procedures, primers, and Illumina sequencing detail were as described in Comeau et al. (2017). Sequences were deposited at NCBI as BioProject with accession ID PRJNA541486.

### Sequencing Data Processing

Quality-filtered, demultiplexed fastq files were processed using Mothur package v. 1.38.1 (Schloss et al., 2011). Sequences were quality-trimmed and reverse complemented. To minimize computational effort, the dataset was reduced to unique sequences, retaining total sequence counts. Sequences were aligned to the V4-V5 region of the 16S rRNA gene sequences from the SILVA v.132 database (Quast et al., 2013). Aligned sequences were kept, and this dataset was again reduced to unique sequences. Further, singletons were removed from the dataset, and the remaining sequences were preclustered if they differed by 1 nucleotide position. Sequences classified as eukaryote, chloroplast, and mitochondria according to SILVA taxonomies were removed. Chimeras were identified and removed using the UCHIME algorithm (Edgar et al., 2011). A distances matrix of clustered operational taxonomic units (OTUs) was created using furthest neighbor method at 97% similarity. Representative sequences of OTUs were retrieved based on the mean distance among the clustered sequences.

### Statistical Analysis

Univariate measures of diversity (observed richness, Chao1, Simpson, and Shannon) were calculated for each sample. A principal coordinate analysis (PCO) based on a Bray–Curtis distance matrix was used to explore for differences in the bacterial community of the studied species over the study period using Primer v7 (Anderson et al., 2008). Bacterial community similarity was compared among samples using Bray–Curtis similarity matrices of relative abundance at OTU level. Statistical differences in the composition of the bacterial community between species and between years were assessed using permutational analysis of variance (PERMANOVA) at class and OTU levels, using “species” and “year” as fixed factors in Primer. Similarity percentage (SIMPER) analysis was also used to determined OTUs that contributed to differences among sponge species using OTU abundance in Primer.

In addition, we analyzed the core community in order to further assess the stability of the microbiome over time in each sponge host. We defined core community as the subset of OTUs that occurred (with a relative abundance >0.01%) in all replicates in a sponge host. Core community similarity was compared among samples based on Bray–Curtis similarity matrices of OTU abundance. Differences in the abundance of core OTUs within sponge hosts were assessed with PERMANOVA using “year” as fixed factor.

Functional pathway profiles were predicted using Tax4Fun, which is a software package that predicts the functional capabilities of bacterial communities based on 16S rRNA OTU abundance data (Aßhauer et al., 2015). This approach has been successfully applied in recent studies (Helber et al., 2019; Lurgi et al., 2019; Steinert et al., 2019). For this analysis, two different formatted datasets were created using the initial OTU table: (a) sponge host and year specific sample groups (*n* = 10 samples) and (b) core (i.e., OTUs present in all host-specific samples), shared (i.e., OTUs present in two different years within each sponge host), and transient (i.e., OTUs only present in one single year) (*n* = 16 samples). All Tax4Fun calculations were performed using the standard parameters (fctProfiling = TRUE, refProfile = “UProC,” shortReadMode = FALSE, normCopyNo = TRUE). To assess the quality of the predictions, the Fraction of Taxonomic units Unexplained (FTU) values for all samples were obtained. The FTU measures the fraction of sequences assigned to taxonomic units that cannot be mapped to organisms in the KEGG database using the Tax4Fun association matrix. Subsequently, STAMP v.2.1.3 (Parks et al., 2014) was used to visualize the KEGG pathways abundance profiles using the PCA and heatmap and clustering functions using Ward’s clustering. In order to test for statistical differences between multiple sample groups we used STAMP to run ANOVA, Games–Howell *post hoc* test, and Benjamini–Hochberg FDR for multiple test corrections. Multivariate tests were performed using the *betadisper*-*permutest*, and *adonis* (i.e., PERMANOVA) functions available in the vegan package v.2.5-3 (Oksanen et al., 2012) in R v.3.5.1 (R Core Team, 2018) utilizing Bray–Curtis distance matrices derived from the available functional pathway profiles using the vegan *vegdist* function. Please note that results from predictive functional profiling using 16S rRNA marker genes can deviate from metagenomic profiling and functional gene annotation. In addition, because of functional overlap, some KOs can be assigned to more than one pathway. Nonetheless, in broad bacterial community ecology surveys, like the present study, predictive functional profiling from 16S rRNA gene sequence data can serve as a complementary and cost-effective metagenomic pre-study.

In total, 6275 KEGG Orthologs were predicted among all sponge samples. Due to the predictive nature of these results and to avoid overinterpretation regarding the presence, absence, and abundance, of individual KOs, we only used the Tax4Fun summarized functional pathway abundance profiles, which resulted *n* = 262 pathways. The predicted functional pathway profiles were reformatted and pooled per (a) sponge-species and sampling year and (b) core-, shared-, and transient-community types (as defined above) per sponge-species for functional analyses (see Supplementary Data Sheet S2). We assume that core OTUs are present in all sponges across larger time spans, whereas shared OTUs are present in sponges from different years, but not as consistently as core OTUs. Finally, we assume that transient OTUs are from seawater and are therefore only present at one time point.

## Results

### Sponge Monitoring and Seawater Temperature Records

During the 24-month period of study, a total of seven sponge individuals representing 11% of the total number of tagged sponges in 2016 were sampled. From the total tagged specimens, three individuals of *Isodictya* sp. were sampled in 2016 and resampled in 2017 and 2018 (*n* = 8 samples). Two individuals of *H. torquata* were resampled in 2018 (*n* = 4 samples). In addition, single specimens of *Mycale (Oxymycale) acerata* and *Tedania* (*Tedaniopsis*) were successfully resampled in 2017 and 2018 (*n* = 3 samples for each individual) (Figure 1). During the study, we observed in a few cases, signs of necrosis on sponges, which were not found during the following season (Figure 1B).

Mean measured seawater temperature in late January/early February 2016 was 0.7 ± 0.8°C, whereas it was 1.2 ± 0.4°C in February 2018 (Supplementary Figure S1). In contrast, temperatures ranging between 2 and 2.9°C were recorded in February 2017 (2.5 ± 0.1°C). Our yearlong record between 2016 and 2017 showed a significant increase in temperature above the normal for more than 25 days, reaching a peak of almost 3°C in mid-February 2017 (Supplementary Figure S1, see also Cárdenas et al., 2018b).

### General Patterns in the Bacterial Community

**FIGURE 2:**
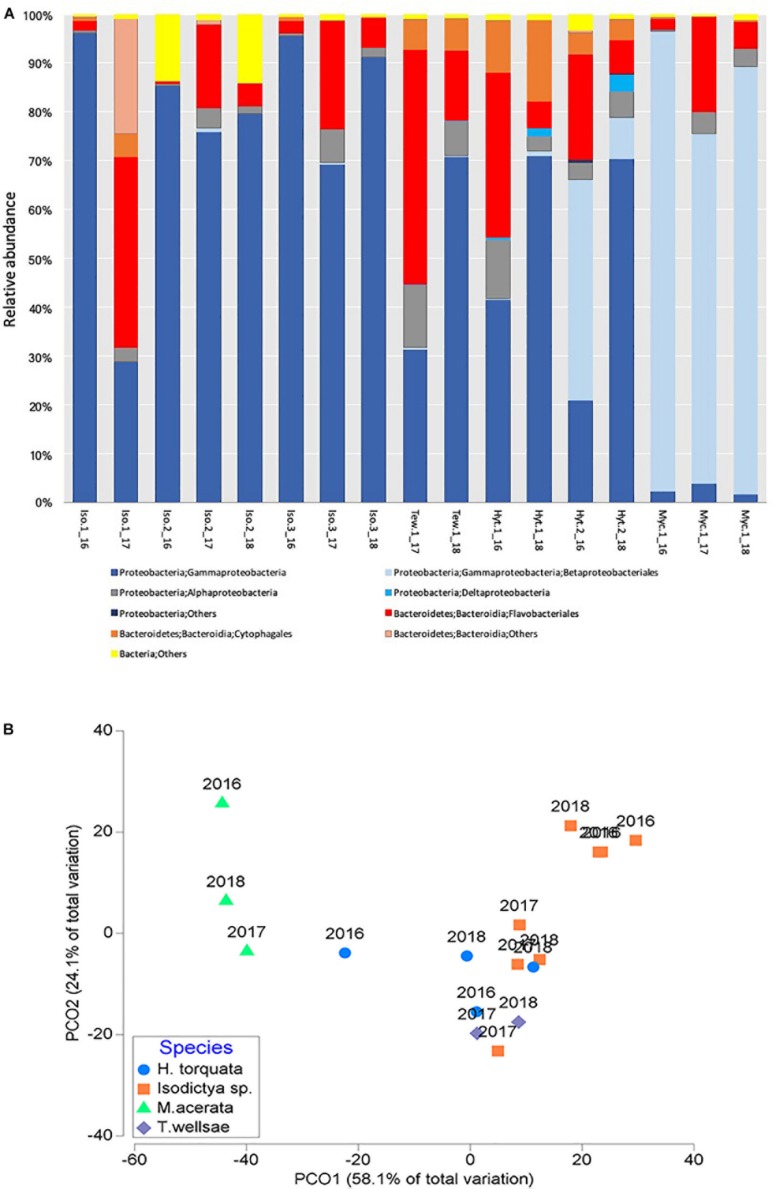
**(A)** Relative abundance of bacterial taxa (at class/order) and **(B)** principal coordinate analysis (PCO) of the bacterial communities associated with four Antarctic sponges monitored during three austral summers over a 24-month period (2016–2018) at Doumer Island, Palmer Archipelago, WAP. Iso, *Isodictya* sp.; Ted, *Tedania* (*Tedaniopsis*) *wellsae*; Hym, *Hymeniacidon torquata*; Myc, *Mycale* (*Oxymycale*) *acerata.*

A total of 17 phyla were found in the four studied sponge species. *H. torquata* presented the highest phylum richness with 14 phyla followed by *M. acerata* with 13, *T. wellsae* and *Isodictya* sp. with 12 phyla each. Bacterial communities of the studied species were dominated by Proteobacteria, followed by Bacteroidetes. Among Proteobacteria, Gammaproteobacteria dominated the bacterial community in all species (Figure 2A).

Results suggest that bacterial communities varied depending on the host when analyzed (*df* = 3,16; *R*^2^ = 0.63, *p* = 0.0001; Figure 2), with a higher level of dissimilarity recorded in *M. acerata* compared with the other sponge species (Figure 2B).

The mean OTU richness was 139.3 ± 90.3 in *H. torquata* and 121.3 ± 71.3 in *Isodictya* sp. The mean OTU richness in samples of *Tedania* (*Tedaniopsis*) *wellsae* was 198.5 ± 31.8, whereas in *M. acerata* the richness was 179.3 ± 30.7. A similar situation was observed for the estimated richness (Chao1). Shannon and Simpson diversity indexes were highly similar between species (Table 1 and Supplementary Figure S2).

**TABLE 1:** Diversity measures of the bacterial communities associated with four Antarctic sponges monitored during three austral summers over a 24-month period (2016–2018) at Doumer Island, Palmer Archipelago, WAP.

**Species**	**Sobs**		**Chao**		**Shannon**		**Simpson**	
				
	**Mean**	**SD**	**Mean**	**SD**	**Mean**	**SD**	**Mean**	**SD**
*Isodictya* sp.	121.3	71.3	125.8	70.6	2.1	0.9	0.3	0.1
*H. torquata*	139.3	90.3	117.8	93.0	2.1	0.4	0.2	0.0
*T. wellsae*	198.5	31.8	201.1	35.2	2.8	0.5	0.1	0.1
*M. acerata*	179.3	30.7	183.1	30.9	1.6	0.4	0.4	0.1

*Values were calculated from all samples of each species collected during the study. The variability of diversity measure for each species over the sampling period is shown in Supplementary Figure S2.*

The permutational multivariate analysis of variance at OTU level show that sponge species host different bacterial communities (*df* = 3,16, *R*^2^ = 0.37, *p* = 0.007). In general, the most abundant OTUs belong to Gammaproteobacteria and most of them were found in all studied species. Eight of the 10 most abundant OTUs belonged to this class (OTUs 1, 2, 3, 4, 7, 8, 9, 10) (Supplementary Table S1). Interestingly, OTU2 (Betaproteobacteriales, EC94) was especially abundant in *M. acerata* (representing 73% of the community), but were present in other species in very low abundances (<0.1%). Bacteroidetes was represented among the 10 most dominant OTUs by OTU5 and OTU6, belonging to Flavobacteriaceae and Cyclobacteriaceae, respectively. These two OTUs were mainly dominant in *H. torquata* and *T. wellsae*. In all comparisons, a few OTUs (between 2 and 5) were responsible for about 50% of the differences between sponge hosts (Supplementary Table S1).

### Temporal Patterns in the Bacterial Community

Overall, the studied bacterial communities analyzed at class level did not change between years (*df* = 2,16, *R*^2^ = 0.1, *p* = 0.1596). This is confirmed by the PCO analysis which showed no evidence of clear temporal patterns in the bacterial communities (Figure 2B).

When analyzed at OTU level, the temporal patterns in the bacterial communities of the studied species also did not showed differences thought time (*df* = 2,16, *R*^2^ = 0.11, *p* = 0.3997). Analyses at OTU level showed similarity values between samples ranging from 61 to 33.7% in samples of *H. torquata. Isodictya* sp. showed highly variable levels of similarity ranging from 67.5 to 4.1%; however, this variability was not consistent across years. Similarity in *M. acerata* showed values between 49.3 and 46.8% between years, whereas in *T. wellsae* it reached 51.6% (Supplementary Figure S3).

**FIGURE 3:**
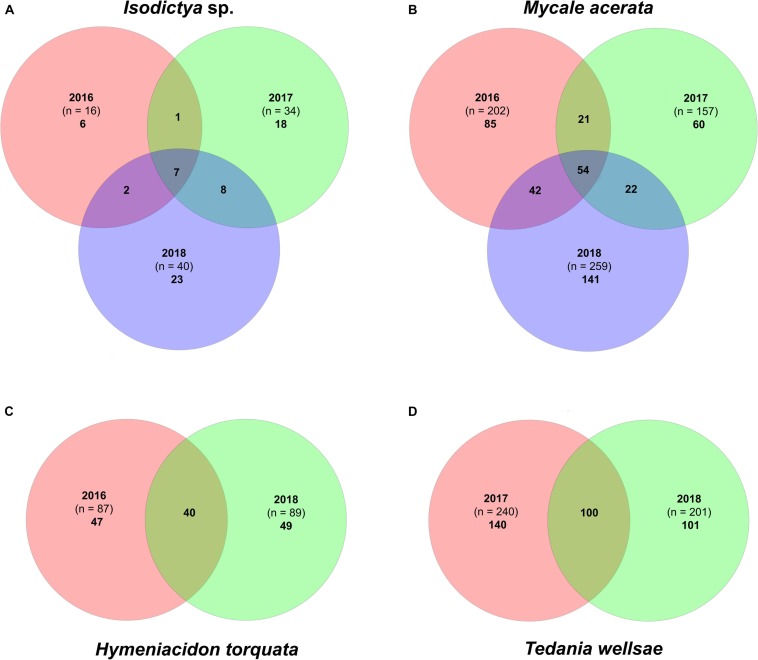
Venn diagram showing the core bacterial communities in **(A)** *Isodictya* sp., **(B)** *Mycale* (*Oxymycale*) *acerata*, **(C)** *Hymeniacidon torquata*, and **(D)** *Tedania* (*Tedaniopsis*) *wellsae*, from sponges monitored during three austral summers over a 24-month period (2016–2018) at Doumer Island, Palmer Archipelago, WAP. Venn diagrams **(B)** and **(C)** are based on a single sponge resampled in different years.

The core bacterial community was represented by a few OTUs that accounted for a high percentage of the total counts (Supplementary Tables S2, S3). *Isodictya* sp. and *H. torquata* were the species that showed larger variability between years compared with the other two (Figure 3). Samples of *Isodictya* sp. from 2017 shared only 8 and 15 OTUs with those from 2016 and 2018, respectively, and only 7 OTUs (representing 44% of the total OTU count) were shared in all years (Figure 3). The core of *H. torquata* constituted 40 OTUs, which represent 77% of the OTU count. In contrast, the core bacterial community of *M. acerata* and *T. wellsae* was comprised by 54 and 100 OTUs, constituting 94 and 96% of the total counts, respectively (Figure 3 and Supplementary Tables S2, S3).

**FIGURE 4:**
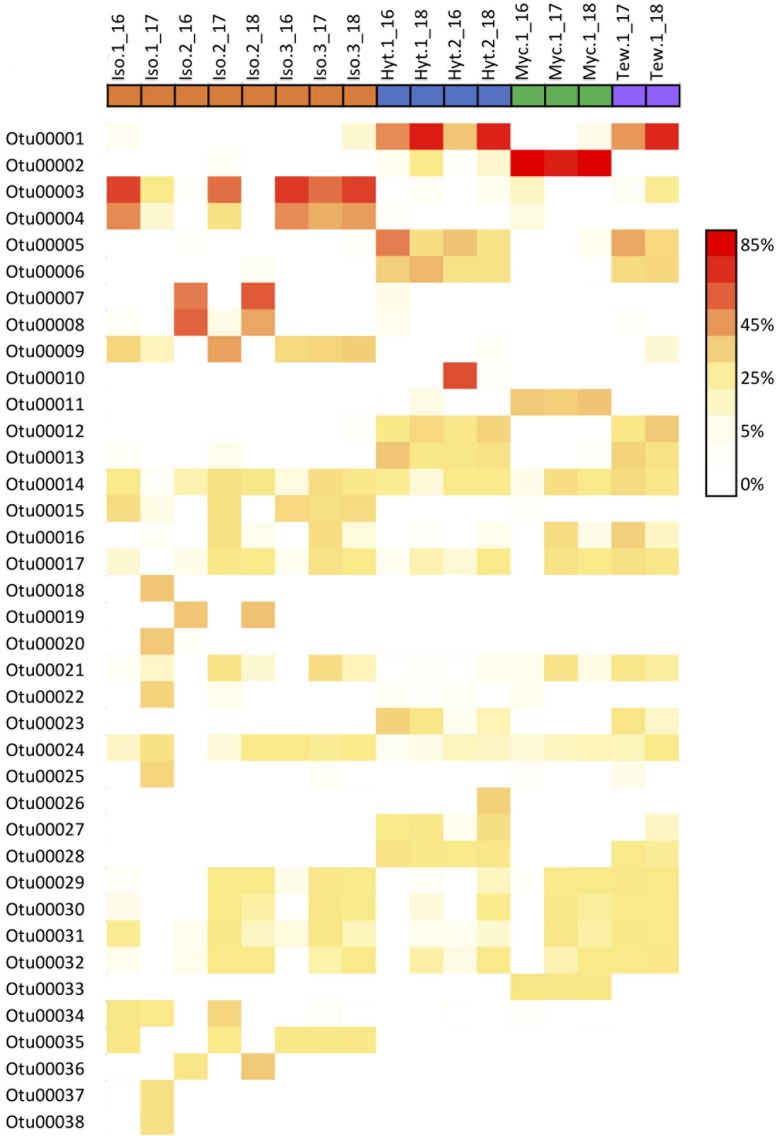
Heatmap showing relative abundances of the 38 most abundant core OTUs in samples of four Antarctic sponges monitored during three austral summers over a 24-month period (2016–2018) at Doumer Island, Palmer Archipelago, WAP.

Overall, the core community within each sponge host showed a high stability over time (Figure 4 and Supplementary Data Sheet S1), averaging 83.5 and 78.2% similarity for *Isodictya* sp. and *H. torquata*, respectively (Tables 2, 3). High mean similarities were also recorded in individuals of *M. acerata* (91.6%) and *T. wellsae* (76.1%) resampled during the study. Statistical analyses (PERMANOVA) confirmed the stability of the core community in *Isodictya* sp. (*df* = 2,7, *p* = 0.8283) with most core OTUs maintaining their abundances over time with the exception of one individual (Iso.2) that showed more variability between years in some core OTUs (Gammaproteobacterial OTUs 3 and 4) (Figure 4). A similar pattern was observed in *H. torquata* (*df* = 1,3, *p* = 0.0506), where most OTUs showed little variation with the exception of OTU10 (Gammaproteobacteria), which showed a particular high representation in one individual (Hyt.2) in 2016 (Figure 4). In the case of the individual of *M. acerata*, the variability was also low, with a betaproteobacterial OTUs (OTU2 and 11) being consistently abundant over time.

**TABLE 2:** Similarity of bacterial core communities in samples of *Isodictya* sp. monitored during three austral summers over a 24-month period (2016–2018) at Doumer Island, Palmer Archipelago, WAP.

	**Iso. 1_16**	**Iso. 1_17**	**Iso. 2_16**	**Iso. 2_17**	**Iso. 2_18**	**Iso. 3_16**	**Iso. 3_17**
Iso.1_17	71.80						
Iso.2_16	71.69	99.43					
Iso.2_17	86.48	81.60	81.49				
Iso.2_18	71.68	98.36	98.92	82.41			
Iso.3_16	98.75	71.26	70.97	85.25	70.49		
Iso.3_17	90.73	76.28	76.17	94.22	77.00	89.54	
Iso.3_18	97.59	72.46	72.36	88.13	73.35	97.00	92.27

**TABLE 3:** Similarity of bacterial core communities in samples of *Hymeniacidon torquata* monitored during three austral summers over a 24-month period (2016–2018) at Doumer Island, Palmer Archipelago, WAP.

	**Hyt.1_16**	**Hyt.1_18**	**Hyt.2_16**
Hyt.1_18	78.24		
Hyt.2_16	76.65	75.62	
Hyt.2_18	73.90	87.67	77.25

### Predicted Functional Profiles

The potential functional roles of host-specific and core, shared, and transient sponge-associated bacterial communities were predicted using Tax4Fun. In the present study, the FTU value (i.e., FTU), as a measure of the quality of the predictions, varied from 0.04 to 0.95. Low FTU values indicate that major fractions of the available OTUs were included in the functional prediction, which further indicates that most OTUs were represented by at least one KEGG organism in the reference data of Tax4Fun. Across all specimens *n* = 9 samples yielded FTU values ≤0.30, *n* = 5 samples yielded FTUs between >0.30 and <0.80, and *n* = 5 samples showed FTU values ≥0.80 (Supplementary Table S4).

**FIGURE 5:**
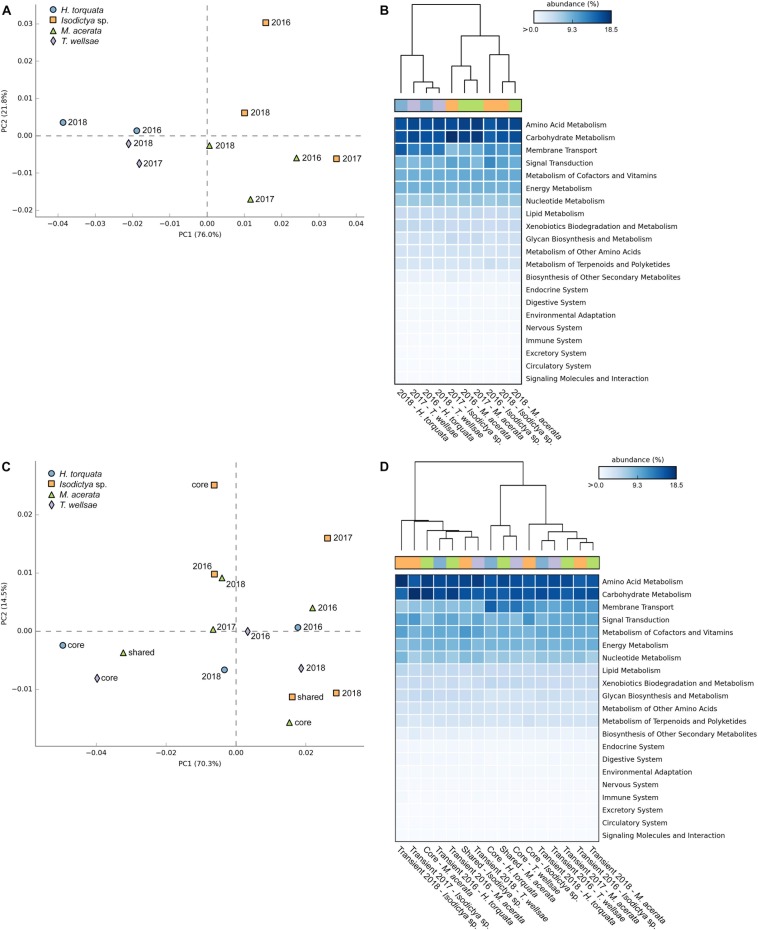
PCA, heatmap, and hierarchical clustering visualizations of the KEGG pathway relative abundance profiles for panels **(A)** and **(B)** the predicted functional pathways among the four sponge species over time, and panels **(C)** and **(D)** the predicted functional pathways of the core, shared, and transient sponge-associated bacterial communities.

First, we analyzed the predicted functional pathway patterns among the bacterial communities of the four sponge species over time (Figures 5A,B). The PERMANOVA using Bray–Curtis distance matrices derived from the predicted functional pathways profile showed that a large fraction of the variance was significantly explained by sponge identity (*df* = 3,6, *R*^2^ = 0.57, *p* = 0.023) while controlling for the year of sampling (strata = Year). The multivariate homogeneity of group variances was not significantly affected by sponge identity (*df* = 3,6, *p* = 0.22), which strengthens the observed significant differences in multivariate variances. Both displayed principal component analysis (PCA) axes clearly separate the individual sponges by taxonomy, with the exception of *Isodictya* sp. from 2017, which was closer to the *M. acerata* group (Figure 5A). The PCA plot shows that most of the variance (76.0%) is explained by the first principal component, whereas the second principal component explains 21.8% (Figure 5A). The heatmap and cluster analysis mostly resembles the observed PCA clustering with two distinct main clusters separating the *H. torquata* and *T. wellsae* individuals from *Isodictya* sp. and *M. acerata* individuals (Figure 5B). Nevertheless, within these clusters, samples appear to be less species specific. In all cases the sampling time does not have an apparent effect on how samples are related to each other (Figures 5A,B). Despite the observed sponge–host-specific patterns, no significant differences were detected between multiple sample groups (i.e., host identity) (*p* > 0.05) after Benjamini–Hochberg FDR corrections for multiple test.

Secondly, we analyzed the predicted functional pathway patterns of the core, shared, and transient sponge-associated bacterial communities (Figures 5C,D). The PERMANOVA showed no significant effect of the community type (i.e., core, shared, transient) on the observed variance (*df* = 4,11, *R*^2^ = 0.29, *p* = 0.53) while controlling for the identity of sponges (strata = Sponge). The multivariate homogeneity of group variances was also not significantly affected by the community type (*df* = 4,11, *p* = 0.77). In case of the core and variable community pathways, the separation by both displayed PCA axes does not reflect the host-identity (Figure 5C). However, the core community functional pathways display lower variability between *H. torquata* and *T. wellsae*. In contrast, the predicted core functions in *Isodictya* sp. and *M. acerata* are clearly separated. Despite the lack of host-related clustering, the transient community pathway functions apparently cluster mostly around the center of both displayed principal components, implying higher similarity among those samples (Figure 5C). However, the close proximity of the samples to the center also suggests that these samples do not possess the same functions as samples, which are further away from the origin. Again, the heatmap and cluster analysis mostly resembles the observed PCA clustering with two distinct main clusters and two sub-clusters separating the second main cluster (Figure 5D). Within these clusters the predicted functional pathways derived from the transient communities mostly cluster together and the shared and core functions from *H. torquata* and *T. wellsae* sharing the smaller sub-cluster. Similar to the host-specific statistical test we observed no significant differences between multiple sample groups (i.e., core, shared, and transient) (*p* > 0.05) after Benjamini–Hochberg FDR corrections for multiple tests.

In both group-specific analyses the most abundant pathways among all sponges belonged mostly to metabolism pathway groups, such as amino acid, carbohydrate, energy, and nucleotide (Figures 5B,D and Supplementary Data Sheet S2). In addition, also pathways related to membrane transport and signal transduction were predicted to be present with relatively high abundances.

## Discussion

Our results showed that studied sponges host different bacterial communities, being dominated by Proteobacteria and Bacteroidetes which is in accordance with previous studies on other Antarctic sponges that has classified them as LMA species (Rodríguez-Marconi et al., 2015; Cárdenas et al., 2018a; Steinert et al., 2019). Studied species were mainly dominated by a few OTUs that constitute large proportions of the community. A similar pattern has been previously described for other species from the Antarctic (Rodríguez-Marconi et al., 2015; Cárdenas et al., 2018a) and other latitudes (Croué et al., 2013; Giles et al., 2013; Simister et al., 2013). In general, the bacterial communities of *H. torquata*, *Isodictya* sp., and *T. wellsae* were dominated by different OTUs belonging mainly to Gammaproteobacteria and Bacteroidia. In contrast, the microbiome of *M. acerata* showed a different pattern, being dominated by a betaproteobacterial OTU (OTU2, EC94). The microbiome of *Isodictya* sp. when compared with the closely related species *Isodictya bentarti*, from South Shetland Islands (Steinert et al., 2019), was remarkably different. While the microbiome of *Isodictya* sp. was dominated by gammaprotebacterial OTUs, *I. bentarti* was mainly dominated by beta- and alphaproteobacterial OTUs. The observed differences in the microbiome of Antarctic sponges coupled with their great phenotypic plasticity in metabolic physiology reported in previous studies, highlights their role as potential key factors in their success in the extreme Antarctic environment (Morley et al., 2016).

The bacterial communities of the four studied sponge species remained stable during the 24-month study, which is in accordance with previous studies that showed that sponges tend to be highly stable in time and also when exposed to environmental variation (Thoms et al., 2003; Erwin et al., 2012; Simister R. et al., 2012; Björk et al., 2013; Pineda et al., 2017; Glasl et al., 2018). However, some studies have detected variability in a few sponges including species of *Hymeniacidon* (Cao et al., 2012; Weigel and Erwin, 2017) which has led to the hypothesis that some lineages of LMA sponge exhibit greater variability over time (Erwin et al., 2015). Although we recorded some variability in a few particular OTUs in one individual of *H. torquata*, overall samples showed a high similarity with no changes in the core community over time, which is similar to the trend of high stability of the microbiome of *Isodictya* sp., which with the exception of one individual that was more variable, individuals tend to show high similarities over time.

As reported in previous studies (e.g., Erwin et al., 2012), the observed stability was driven by the persistent presence of dominant core OTUs, that despite being small in numbers, accounted for the majority of the total abundance. The core members of the bacterial communities seemed to remain stable through time and seasonal fluctuations in abiotic factors such as temperature, which is an important environmental driver structuring the sponge microbiome (Pita et al., 2018; Lurgi et al., 2019). The bacterial communities of the studied species remain stable even after being naturally exposed to above normal temperatures (3°C) and more importantly to significant warming rates recorded in summer 2017 (0.11–0.15°C day^–1^compared to 0.04 day^–1^ recorded in normal periods, Cárdenas et al., 2018b), which can have a significant impact in the mechanisms dictating survival limits of Antarctic benthic organisms (Peck, 2018). In this regard, research on temperate and tropical sponges suggest that while some species have the ability to cope with thermal stress, at least over a narrow temperature range (Simister R. et al., 2012; Pineda et al., 2016), in other cases it has been reported that changes in water temperature can disrupt the microbiome (e.g., Webster et al., 2008; Fan et al., 2013; Ramsby et al., 2018), altering the structure and the diversity of the microbiome with increases in rare and/or opportunistic microorganisms, often affecting host health (Lemoine et al., 2007; Webster et al., 2008; Luter et al., 2012; Fan et al., 2013). The slight variations in the abundance of a few OTUs in some species were not correlated with a particular period or temperature increase; hence, suggesting that other factors rather than temperature influenced the observed variation. However, since the strength and/or duration of perturbation plays a significant role determining the ability of the holobiont to cope with disturbance (see Pita et al., 2018), it is possible that the seawater temperatures recorded in our study might be within the range where the holobionts are still able to respond and maintain a stable state, hence further warming may produce alterations to their stability. Our temporal study has a limitation that needs to be acknowledged. The limited access to the study area in other seasons than summer is one of the main challenges associated with working in Antarctica (Kennicutt et al., 2016). This certainly limited our possibilities of increasing the frequency of sampling that could potentially provide more insights on the response of the microbiome to changes in productivity occurring in the WAP during winter/summer transition (reviewed by Schofield et al., 2018). Similar studies based on repeated sampling of individuals have found contrasting results. For instance, a study of *Mycale hentscheli* from New Zealand (Anderson et al., 2010) reported that similarities in the microbiome were variable within and between individuals, after monitoring sponge individuals six times over a 21-month period. In contrast, another study of three *Ircinia* species monitored six sampling times over a 15-month period in the NW Mediterranean Sea reported high stability and no clear shifts in their microbiome, despite significant seasonal variation in seawater temperature and irradiance (Erwin et al., 2012). This suggest that although periodicity is important when studying temporal variation on the sponge microbiome, their patterns are highly dependent on the sponge host and hence, more variable microbiomes are not necessarily correlated with sponges inhabiting more dynamic environments.

In addition, although a large number of sponges were tagged in 2016, the majority of the specimens were lost in the following sampling times. This is explained by the strong influence of ice scour in the area and in the WAP in general (Barnes, 2017), which produce mass mortality of benthic organisms, hence creating new areas for colonization that are characterized by the presence of small sessile organisms (e.g., small/young sponges). Barnes (2017) provided insights of the dramatic effects of ice scour in shallow areas around the WAP, where in some areas such as Ryder Bay (Adelaide Island), about 25% of the substrate is hit by icebergs each year. Although this type of estimation was not carried out in our study area, the impact of ice scour was often observed, even increasing in late summer (February) 2016, affecting our study site by removing sponges and moorings containing temperature data-loggers. This shows the difficulties of working in such an extreme environment, yet highlights the importance of the results presented here, as they provide the first insights on the temporal patterns of bacterial communities associated with Antarctic sponges.

Several sponge-associated microbiota studies applied 16S rRNA gene-related functional prediction to gain first insights into the functional diversity and potential of the bacterial assemblages (de Voogd et al., 2015; Weigel and Erwin, 2017; Cleary et al., 2018a, b; Helber et al., 2019; Steinert et al., 2019). Despite the scarcity of actual ‘omics-based bacterial functional surveys (i.e., meta-genomics, -transcriptomics, or -proteomics) comprising multiple sponge-species, the overall available results are supporting the functional host-specificity (Fan et al., 2012; Rua et al., 2015; Borchert et al., 2016; Horn et al., 2016; Ryu et al., 2016). Even though host-specific differences were apparent, core functions (i.e., stress resistance, phage defense, denitrification, ammonium oxidation, horizontal gene transfer, or biosynthesis of cofactors, vitamins, and prosthetic groups) were observed among phylogenetically divergent sponge holobionts (Fan et al., 2012; Rua et al., 2015; Li et al., 2016). The concept of core functions implies functional equivalence, meaning that phylogenetically divergent bacteria can inhabit analogous niches in different sponge species and still exhibit similar functions. However, the functional stability observed here for multiple sponge hosts over time still needs to be validated by future ’omics-based studies. Considering the lack of ’omics-based sponge holobiont surveys that take temporal or environmental variation into account, comparisons of our results with other studies are not yet feasible. Here we observed that the predicted functional temporal cores display similar relationships to each other as the annual species-specific predicted functional profiles. This is in contrast to the predicted transient functional cores, which are more similar to each other than the related functional cores. These patterns indicate the importance of the species-specific core symbionts, which appear to be responsible for the predicted species-specific core functions that are stable over time and potential environmental variation. Nonetheless, these predicted species-specific functional cores should not be confused with the core functions that are present among phylogenetically different holobionts, such as sponges (Fan et al., 2012; Rua et al., 2015) or different green algae species (Roth-Schulze et al., 2018). In this regard, the potential role played by their symbionts providing capacity to sponge hosts to cope with rapid environmental change along with their great phenotypic plasticity in metabolic physiology may explain their success in Antarctic environments and also may improve the chances of more sponge species to become “winners” under the climate change scenario. Nevertheless, we want to emphasize again that functional metabolic predictions are no substitute for metabolic gene or gene cluster analyses. Therefore, future metagenomics or whole genome sequencing surveys are required to validate if sponge-associated bacterial symbionts possess the pathways and patterns predicted in the present study.

## Conclusion

The monitoring of tagged sponges over a 24-month period allowed us to characterize for the first time the temporal patterns in diversity and structure in the bacterial communities of a taxonomically diverse set of Antarctic sponges. We have also provided information on potential temporal functional patterns between and among the four sponge species. Our results showed no consistent variation, indicating that the bacterial community of the studies species show a high temporal stability, even after being naturally exposed to significant warming rates during the austral summer 2017. The high stability was driven by the persistent presence of dominant core OTUs, that was comprised by a small number of OTUs that accounted for the majority of the total abundance of the community. Future studies may confirm the resilience of the microbiome of Antarctic sponges to further warming as it has been projected for the WAP.

## Data Availability Statement

The datasets generated for this study can be found in the sequences deposited at NCBI as BioProject with accession ID PRJNA541486.

## Author Contributions

CC conceived and carried out the field study. AF and GS carried out the sequence analyses. CC, AF, and GS carried out the statistical analyses, and wrote the manuscript with contributions of RR and MG-A refining the text. All authors read and approved the final manuscript.

## Conflict of Interest

The authors declare that the research was conducted in the absence of any commercial or financial relationships that could be construed as a potential conflict of interest.

## Footnotes

1 http://cgeb-imr.ca/

## Supplementary Material

FIGURE S1 Daily mean seawater temperature recorded at Doumer Island, Palmer Archipelago, WAP. Bars indicate sampling times.

FIGURE S2 Observed richness (Sobs), estimated richness (Chao1), Simpson and Shannon diversity indexes of the bacterial communities associated with four Antarctic sponges monitored during three austral summers over a 24-month period (2016–2018) at Doumer Island, Palmer Archipelago, WAP.

FIGURE S3 Similarity of the bacterial communities associated with four Antarctic sponges monitored during three austral summers over a 24-month period (2016–2018) at Doumer Island, Palmer Archipelago, WAP. Heatmap illustrates Bray-Curtis similarities expressed as percentage.

TABLE S1 Most discriminant bacterial OTU from samples of four Antarctic sponges according to SIMPER analysis. Selected OTUs contributed to 50% of the dissimilarities between sponge species.

TABLE S2 Taxonomy of the most dominant bacterial OTUs.

TABLE S3 Thirty most dominant bacterial OTUs of the temporal core community recorded in each host species.

TABLE S4 Sample information and fraction of unmapped Tax4Fun OTUs (FTU).

DATA SHEET S1 Relative abundances of core OTUs in samples of four Antarctic sponges monitored during three austral summers over a 24-month period (2016–2018) at Doumer Island, Palmer Archipelago, WAP.

DATA SHEET S2 Formatted as STAMP and R input files.
