# Supplementary material for: Temporal Stability of Bacterial Communities in Antarctic Sponges
Source: Front Microbiol. 2019 Nov 22;10:2699. doi: 10.3389/fmicb.2019.02699 (PMC6883807; doi:10.3389/fmicb.2019.02699)
Supplement: FIGURE S1 — Daily mean seawater temperature recorded at Doumer Island, Palmer Archipelago, WAP. Bars indicate sampling times. [file Presentation_1.pdf]

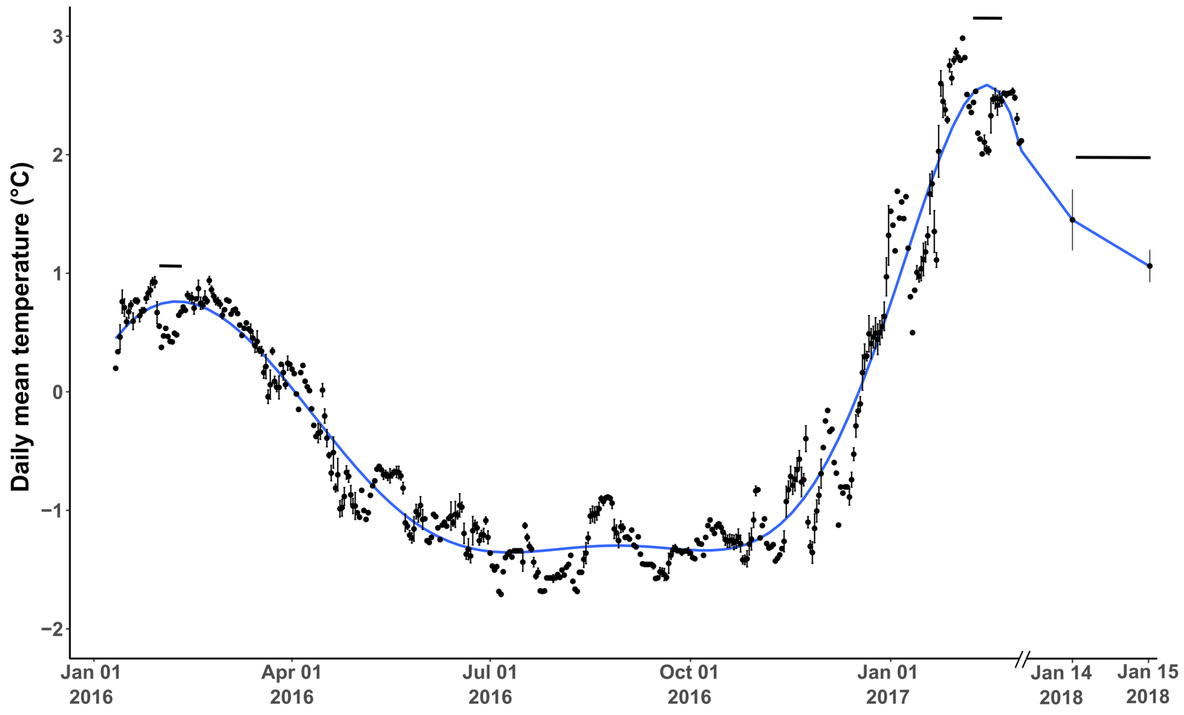

Suppl. Fig. 1. Daily mean seawater temperature recorded at Doumer Island, Palmer Archipelago, WAP. Bars indicate sampling times.

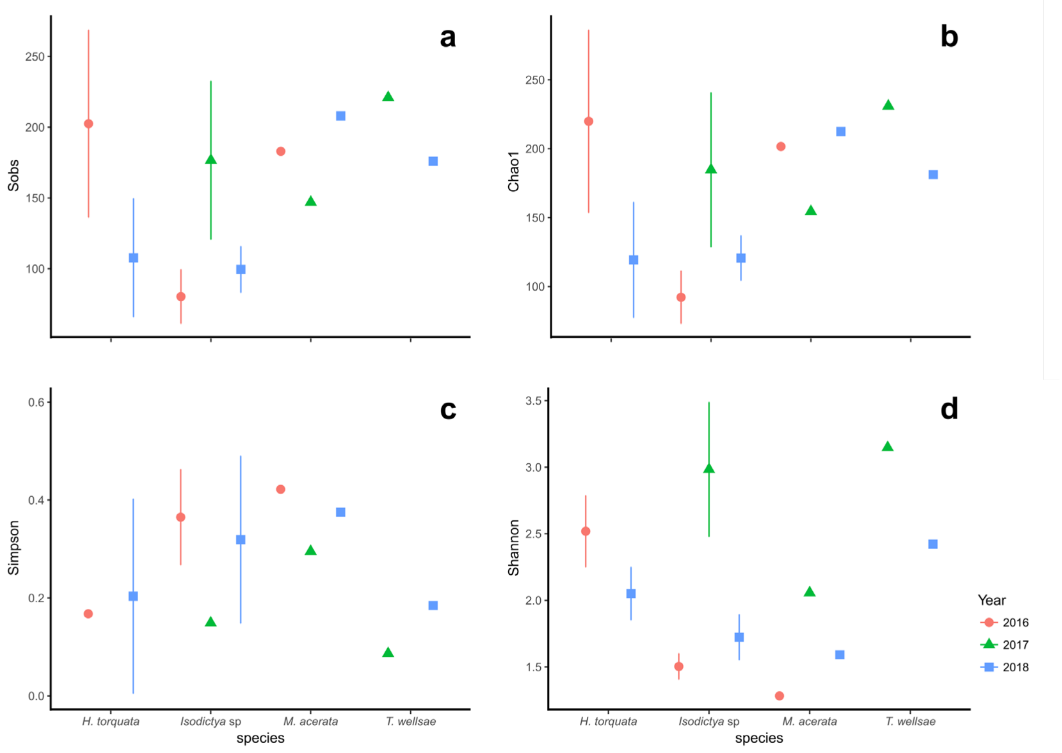

Suppl. Fig. 2. Observed richness (Sobs), estimated richness (Chao1), Simpson and Shannon diversity indexes of the bacterial communities associated with four Antarctic sponges monitored during three austral summers over a 24-month period (2016-2018) at Doumer Island, Palmer Archipelago, WAP.

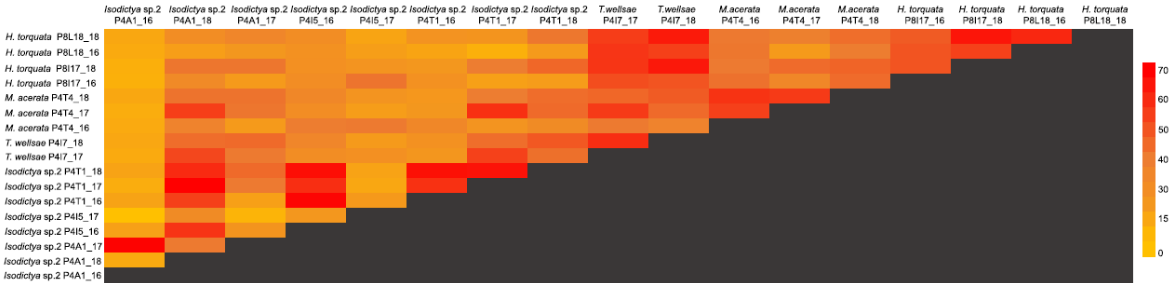

Suppl. Fig. 3. Similarity of the bacterial communities associated with four Antarctic sponges monitored during three austral summers over a 24-month period (2016-2018) at Doumer Island, Palmer Archipelago, WAP. Heatmap illustrates Bray-Curtis similarities expressed as percentage.
