# Supplementary material for: Temporal Stability of Bacterial Communities in Antarctic Sponges
Source: Front Microbiol. 2019 Nov 22;10:2699. doi: 10.3389/fmicb.2019.02699 (PMC6883807; doi:10.3389/fmicb.2019.02699)
Supplement: TABLE S1 — Most discriminant bacterial OTU from samples of four Antarctic sponges according to SIMPER analysis. Selected OTUs contributed to 50% of the dissimilarities between sponge species. [file Table_1.docx]

**Supplementary Table 1.** Most discriminant bacterial OTU from samples of four Antarctic sponges according to SIMPER analysis. Selected OTUs contributed to 50% of the dissimilarities between sponge species.

| Groups *Isodictya* sp. & *T. wellsae* | Average dissimilarity = 93.31 | | |  |  |  |
| --- | --- | --- | --- | --- | --- | --- |
|  | *Isodictya* sp. | *T. wellsae* |  |  |  |  |
| OTU | Av.Abund | Av.Abund | Av.Diss | Diss/SD | Contrib% | Cum.% |
| Otu00001 (Gammaproteobacteria unclassified) | 0.03 | 40.89 | 20.43 | 2.33 | 21.89 | 21.89 |
| Otu00003 (Gammaproteobacteria unclassified) | 28.21 | 0.21 | 14.05 | 1.22 | 15.05 | 36.94 |
| Otu00005 (Flavobacteriaceae;Wenyingzhuangia) | 0.01 | 12.73 | 6.36 | 1.76 | 6.82 | 43.76 |
| Otu00004 (Gammaproteobacteria unclassified) | 12.51 | 0.02 | 6.25 | 1 | 6.7 | 50.46 |
| Groups *Isodictya* sp. & *M. acerata* | Average dissimilarity = 95.73 | | |  |  |  |
|  | *Isodictya* sp. | *M. acerata* |  |  |  |  |
| OTU | Av.Abund | Av.Abund | Av.Diss | Diss/SD | Contrib% | Cum.% |
| Otu00002 (Betaproteobacteriales;EC94) | 0.01 | 72.3 | 36.14 | 8.31 | 37.76 | 37.76 |
| Otu00003 (Gammaproteobacteria unclassified) | 28.21 | 0.08 | 14.08 | 1.23 | 14.71 | 52.47 |
| Groups *T. wellsae* & *M. acerata* | Average dissimilarity = 92.00 | | |  |  |  |
|  | *T. wellsae* | *M. acerata* |  |  |  |  |
| OTU | Av.Abund | Av.Abund | Av.Diss | Diss/SD | Contrib% | Cum.% |
| Otu00002 (Betaproteobacteriales;EC94) | 0.01 | 72.3 | 36.15 | 7.75 | 39.29 | 39.29 |
| Otu00001 (Gammaproteobacteria unclassified) | 40.89 | 0.04 | 20.43 | 2.2 | 22.2 | 61.5 |
| Groups *Isodictya* sp. & *H. torquata* | Average dissimilarity = 97.82 | | |  |  |  |
|  | *Isodictya* sp. | *H. torquata* |  |  |  |  |
| OTU | Av.Abund | Av.Abund | Av.Diss | Diss/SD | Contrib% | Cum.% |
| Otu00001 (Gammaproteobacteria unclassified) | 0.03 | 39.97 | 19.97 | 1.81 | 20.41 | 20.41 |
| Otu00003 (Gammaproteobacteria unclassified) | 28.21 | 0.03 | 14.09 | 1.24 | 14.41 | 34.82 |
| Otu00004 (Gammaproteobacteria unclassified) | 12.51 | 0.02 | 6.25 | 1.01 | 6.39 | 41.21 |
| Otu00005 (Flavobacteriaceae;Wenyingzhuangia) | 0.01 | 12.26 | 6.12 | 1.04 | 6.26 | 47.47 |
| Otu00010 (Betaproteobacteriales unclassified) | 0 | 11.05 | 5.53 | 0.57 | 5.65 | 53.12 |
| Groups *T. wellsae* & *H. torquata* | Average dissimilarity = 48.68 | | |  |  |  |
|  | *T. wellsae* | *H. torquata* |  |  |  |  |
| OTU | Av.Abund | Av.Abund | Av.Diss | Diss/SD | Contrib% | Cum.% |
| Otu00001 (Gammaproteobacteria unclassified) | 40.89 | 39.97 | 10.9 | 1.21 | 22.4 | 22.4 |
| Otu00005 (Flavobacteriaceae;Wenyingzhuangia) | 12.73 | 12.26 | 5.6 | 1.37 | 11.5 | 33.9 |
| Otu00010 (Betaproteobacteriales unclassified) | 0 | 11.05 | 5.53 | 0.54 | 11.35 | 45.25 |
| Otu00006 (Cyclobacteriaceae;Ekhidna) | 5.54 | 7.18 | 2.26 | 1.44 | 4.64 | 49.89 |
| Otu00012 (Gammaproteobacteria unclassified) | 5.61 | 3.72 | 2.1 | 1.34 | 4.3 | 54.19 |
| Groups *M. acerata* & *H. torquata* | Average dissimilarity = 97.00 | | |  |  |  |
|  | *M. acerata* | *H. torquata* |  |  |  |  |
| OTU | Av.Abund | Av.Abund | Av.Diss | Diss/SD | Contrib% | Cum.% |
| Otu00002 (Betaproteobacteriales;EC94) | 72.3 | 0.28 | 36.01 | 8.09 | 37.13 | 37.13 |
| Otu00001 (Gammaproteobacteria unclassified) | 0.04 | 39.97 | 19.97 | 1.76 | 20.59 | 57.71 |
