# Supplementary material for: Temporal Stability of Bacterial Communities in Antarctic Sponges
Source: Front Microbiol. 2019 Nov 22;10:2699. doi: 10.3389/fmicb.2019.02699 (PMC6883807; doi:10.3389/fmicb.2019.02699)
Supplement: TABLE S2 — Taxonomy of the most dominant bacterial OTUs. [file Table_2.docx]

Supplementary Table 2. Taxonomy of the most dominant bacterial OTUs

| OTU | Size | Taxonomy |  |  |  |  |  |  |
| --- | --- | --- | --- | --- | --- | --- | --- | --- |
| Otu001 | 48005 | Proteobacteria; Gammaproteobacteria; Gammaproteobacteria_unclassified | | | | | | |
| Otu002 | 46105 | Proteobacteria; Gammaproteobacteria; Betaproteobacteriales; EC94 | | | | | | |
| Otu003 | 33194 | Proteobacteria; Gammaproteobacteria; Gammaproteobacteria_unclassified | | | | | | |
| Otu004 | 14659 | Proteobacteria; Gammaproteobacteria; Gammaproteobacteria_unclassified | | | | | | |
| Otu005 | 10878 | Bacteroidetes; Bacteroidia; Flavobacteriales; Flavobacteriaceae; Wenyingzhuangia | | | | | | |
| Otu006 | 7304 | Bacteroidetes; Bacteroidia ;Cytophagales; Cyclobacteriaceae; Ekhidna | | | | | | |
| Otu011 | 6238 | Proteobacteria; Gammaproteobacteria; Betaproteobacteriales | | | | | | |
| Otu012 | 5876 | Proteobacteria; Gammaproteobacteria; Gammaproteobacteria_unclassified | | | | | | |
| Otu013 | 4703 | Proteobacteria; Alphaproteobacteria; Parvibaculales; PS1_clade; PS1_clade_ge | | | | | | |
| Otu014 | 3878 | Bacteroidetes; Bacteroidia; Flavobacteriales; Flavobacteriaceae; Polaribacter_1 | | | | | | |
| Otu015 | 3273 | Proteobacteria;G ammaproteobacteria; Gammaproteobacteria_unclassified | | | | | | |
| Otu016 | 3089 | Bacteroidetes; Bacteroidia; Flavobacteriales;Flavobacteriaceae; Polaribacter(75) | | | | | | |
| Otu017 | 2697 | Bacteroidetes; Bacteroidia; Flavobacteriales; Flavobacteriaceae; Polaribacter_1 | | | | | | |
| Otu021 | 1966 | Bacteroidetes; Bacteroidia; Flavobacteriales; Flavobacteriaceae; Ulvibacter | | | | | | |
| Otu022 | 1735 | Proteobacteria; Gammaproteobacteria; Oceanospirillales; Nitrincolaceae; Profundimonas | | | | | | |
| Otu023 | 1583 | Proteobacteria; Gammaproteobacteria; Thiomicrospirales; Thioglobaceae | | | | | | |
| Otu024 | 1531 | Bacteroidetes; Bacteroidia; Flavobacteriales; Flavobacteriaceae | | | | | | |
| Otu027 | 1249 | Proteobacteria; Deltaproteobacteria; Bdellovibrionales; Bdellovibrionaceae; Bdellovibrio | | | | | | |
| Otu029 | 1228 | Bacteroidetes; Bacteroidia; Flavobacteriales; Cryomorphaceae; uncultured | | | | | | |
| Otu030 | 1222 | Proteobacteria; Alphaproteobacteria; SAR11_clade; Clade_I; Clade_Ia | | | | | | |
| Otu031 | 1215 | Proteobacteria; Gammaproteobacteria; Oceanospirillales; Nitrincolaceae; uncultured | | | | | | |
| Otu061 | 391 | Proteobacteria; Alphaproteobacteria; Rhodobacterales; Rhodobacteraceae | | | | | | |
| Otu065 | 377 | Bacteroidetes; Bacteroidia; Flavobacteriales; Flavobacteriaceae; uncultured | | | | | | |
| Otu068 | 342 | Bacteroidetes; Bacteroidia; Flavobacteriales; Flavobacteriaceae; Aurantivirga | | | | | | |
| Otu088 | 203 | Bacteroidetes; Bacteroidia; Flavobacteriales; Flavobacteriaceae | | | | | | |
| Otu090 | 202 | Bacteroidetes; Bacteroidia; Flavobacteriales; Flavobacteriaceae; Maritimimonas | | | | | | |
| Otu0126 | 114 | Proteobacteria; Gammaproteobacteria; Thiotrichales; Thiotrichaceae; Cocleimonas | | | | | | |
