# Supplementary material for: Temporal Stability of Bacterial Communities in Antarctic Sponges
Source: Front Microbiol. 2019 Nov 22;10:2699. doi: 10.3389/fmicb.2019.02699 (PMC6883807; doi:10.3389/fmicb.2019.02699)
Supplement: TABLE S3 — Thirty most dominant bacterial OTUs of the temporal core community recorded in each host species. [file Table_3.docx]

Supplementary Table 3. Thirty most dominant bacterial OTUs of the temporal core community recorded in each host species.

| Species | Core count | OTULabel | |  |  |  |  |  |  |  |  |  |  |  |  |  |
| --- | --- | --- | --- | --- | --- | --- | --- | --- | --- | --- | --- | --- | --- | --- | --- | --- |
| *Isodictya* sp. | 50807 | Otu003 | Otu004 | Otu014 | Otu017 | Otu031 | Otu032 | Otu068 |  |  |  |  |  |  |  |  |
| *M. acerata* | 58764 | Otu001 | Otu002 | Otu003 | Otu006 | Otu011 | Otu014 | Otu016 | Otu017 | Otu021 | Otu024 | Otu029 | Otu030 | Otu031 | Otu033 | Otu034 |
|  |  | Otu039 | Otu041 | Otu043 | Otu056 | Otu057 | Otu061 | Otu065 | Otu068 | Otu073 | Otu080 | Otu083 | Otu088 | Otu090 | Otu126 | |
| *T. wellsae* | 36882 | Otu001 | Otu002 | Otu003 | Otu004 | Otu005 | Otu006 | Otu008 | Otu009 | Otu012 | Otu013 | Otu014 | Otu015 | Otu016 | Otu017 | Otu021 |
|  |  | Otu023 | Otu024 | Otu027 | Otu028 | Otu029 | Otu030 | Otu031 | Otu032 | Otu061 | Otu063 | Otu065 | Otu067 | Otu068 | Otu126 | |
| *H. torquata* | 44360 | Otu001 | Otu002 | Otu005 | Otu006 | Otu012 | Otu013 | Otu014 | Otu017 | Otu021 | Otu022 | Otu023 | Otu024 | Otu027 | Otu028 | Otu029 |
|  |  | Otu030 | Otu031 | Otu040 | Otu046 | Otu058 | Otu061 | Otu065 | Otu078 | Otu081 | Otu083 | Otu085 | Otu088 | Otu090 | Otu126 | |
