# Supplementary material for: Temporal Stability of Bacterial Communities in Antarctic Sponges
Source: Front Microbiol. 2019 Nov 22;10:2699. doi: 10.3389/fmicb.2019.02699 (PMC6883807; doi:10.3389/fmicb.2019.02699)
Supplement: TABLE S4 — Sample information and fraction of unmapped Tax4Fun OTUs (FTU). [file Table_4.DOCX]

Supplementary Table 4. Sample information and fraction of unmapped Tax4Fun OTUs (FTU).

| **Samples** | **Sponge** | **Year** | **FTU** |
| --- | --- | --- | --- |
| P4A1_16 | *Isodictya* sp. | 2016 | 0.15 |
| P4A1_18 | *Isodictya* sp. | 2018 | 0.24 |
| P4AI_17 | *Isodictya* sp. | 2017 | 0.69 |
| P4I5_16 | *Isodictya* sp. | 2016 | 0.92 |
| P4I5_17 | *Isodictya* sp. | 2017 | 0.25 |
| P4I7_17 | *Tedania wellsae* | 2017 | 0.30 |
| P4I7_18 | *Tedania wellsae* | 2018 | 0.14 |
| P4T1_16 | *Isodictya* sp. | 2016 | 0.95 |
| P4T1_17 | *Isodictya* sp. | 2017 | 0.69 |
| P4T1_18 | *Isodictya* sp. | 2018 | 0.91 |
| P4T4_16 | *Mycale acerata* | 2016 | 0.95 |
| P4T4_17 | *Mycale acerata* | 2017 | 0.74 |
| P4T4_18 | *Mycale acerata* | 2018 | 0.89 |
| P8I17_16 | *Hymeniacidon torquata* | 2016 | 0.66 |
| P8I17_18 | *Hymeniacidon torquata* | 2018 | 0.17 |
| P8L12_18 | *Hymeniacidon torquata* | 2018 | 0.21 |
| P8L17_18 | *Hymeniacidon torquata* | 2018 | 0.04 |
| P8L18_16 | *Hymeniacidon torquata* | 2016 | 0.46 |
| P8L18_18 | *Hymeniacidon torquata* | 2018 | 0.23 |
